# Built environment is a key driver of cardiometabolic health in two Indigenous groups undergoing rapid lifestyle change

**DOI:** 10.1093/emph/eoag007

**Published:** 2026-04-22

**Authors:** Marina M Watowich, Audrey M Arner, Selina Wang, Echwa John, John C Kahumbu, Patriciah Kinyua, Anjelina Lopurudoi, Francis Lotukoi, Charles M Mwai, Benjamin Muhoya, Boniface Mukoma, Kar Lye Tam, Tan Bee Ting A/P Tan Boon Huat, Michael Gurven, Yvonne A L Lim, Dino Martins, Sospeter Ngoci Njeru, Kee-Seong Ng, Vivek V Venkataraman, Ian J Wallace, Julien F Ayroles, Thomas S Kraft, Amanda J Lea

**Affiliations:** Department of Biological Sciences, Vanderbilt University, Nashville, TN, USA; Department of Biological Sciences, Vanderbilt University, Nashville, TN, USA; Department of Biological Sciences, Vanderbilt University, Nashville, TN, USA; Turkana Health and Genomics Project, Kenya; Turkana Health and Genomics Project, Kenya; Turkana Health and Genomics Project, Kenya; Centre for Community Driven Research, Kenya Medical Research Institute, Nairobi, Kenya; Turkana Health and Genomics Project, Kenya; Turkana Health and Genomics Project, Kenya; Turkana Health and Genomics Project, Kenya; Centre for Community Driven Research, Kenya Medical Research Institute, Nairobi, Kenya; Turkana Health and Genomics Project, Kenya; Department of Integrative Biology, University of California Berkeley, Berkeley, CA, USA; Turkana Health and Genomics Project, Kenya; Department of Parasitology, Universiti Malaya, Kuala Lumpur, Malaysia; Department of Parasitology, Universiti Malaya, Kuala Lumpur, Malaysia; Department of Anthropology, University of California Santa Barbara, Santa Barbara, CA, USA; Department of Parasitology, Universiti Malaya, Kuala Lumpur, Malaysia; Turkana Health and Genomics Project, Kenya; Turkana Basin Institute, Stony Brook University, Stony Brook, NY, USA; Centre for Community Driven Research, Kenya Medical Research Institute, Nairobi, Kenya; Department of Medicine, Universiti Malaya, Kuala Lumpur, Malaysia; Department of Anthropology and Archaeology, University of Calgary, Calgary, AB, Canada; Department of Anthropology, University of New Mexico, Albuquerque, NM, USA; Department of Integrative Biology, University of California Berkeley, Berkeley, CA, USA; Department of Anthropology, University of Utah, Salt Lake City, UT, USA; Department of Biological Sciences, Vanderbilt University, Nashville, TN, USA

**Keywords:** evolutionary mismatch, industrialization, Turkana, Orang Asli, cardiometabolic health

## Abstract

**Background:**

Globally, subsistence-level societies are experiencing rapid urbanization and concomitant increases in cardiometabolic diseases. Generalized measures to quantify lifestyle transitions will facilitate the identification of the most potent drivers of changing health within and between populations, enabling the identification of vulnerable communities, and aiding in the creation of effective policies to minimize disease.

**Methods:**

We developed ten scales *a priori* to quantify unique facets of lifestyle (e.g. urban infrastructure, market integration) from cross-sectional data in two Indigenous, transitioning subsistence groups undergoing rapid change in very different ecological and sociopolitical contexts: Turkana pastoralists of northwest Kenya (*n* = 3692) and Orang Asli mixed subsistence practitioners of Peninsular Malaysia (*n* = 1119). We tested the extent to which these lifestyle scales predicted 16 measures of cardiometabolic health in each group. We also used factor analysis to decompose lifestyle data *post hoc* into salient axes, sensitivity analyses to identify the most important drivers of health, and sex-stratified analyses to investigate whether facets of lifestyle differentially impacted cardiometabolic health among males and females.

**Results:**

Cardiometabolic health was best predicted by measures that quantified urban infrastructure and market-derived material wealth compared to metrics encompassing diet, mobility, or acculturation, and these results were highly consistent across Turkana and Orang Asli and across sexes. Factor analysis results were also highly consistent between the two groups, revealing that lifestyle variation decomposes into two distinct axes–representing the built environment and diet–which change at different paces and have different relationships with health.

**Conclusion:**

Our analyses revealed surprising generalizability: in both the Turkana and Orang Asli, measures of local infrastructure and built environment better predicted cardiometabolic health than other facets of lifestyle that are seemingly more proximate to health, such as diet. We hypothesize that this is because the built environment impacts unmeasured proximate drivers like physical activity and broader access to market goods, and because it serves as a proxy of duration of market integration. Our results support the usage of relatively simple and easy to characterize features of the built environment as a cross-cultural tool in the investigation of lifestyle impacts on cardiometabolic health.

**Lay Summary:**

Worldwide, Indigenous and subsistence-level societies are undergoing rapid urbanization, industrialization, and market integration. To facilitate comparative and within-population analyses of resulting changes in health, we developed ten scales that quantify different features of lifestyle change that occur during this transition. We find that cardiovascular and metabolic health were most strongly explained by measures of the built environment, and these findings were highly consistent across two groups–the Turkana of Kenya and Orang Asli of Peninsular Malaysia–living in highly different environments and experiencing distinct pathways to industrialization.

## INTRODUCTION

In low- and middle-income countries (LMIC) around the world, many populations are transitioning from small-scale, subsistence-level practices to more industrialized, urban, and market integrated lifestyles. These changes are multifaceted and include integration into cash-dependent market economies (i.e. market integration), adoption of practices from neighboring or dominant cultures (i.e. acculturation), and increased access to urban infrastructure (i.e. urbanization), resulting in drastic dietary, nutritional, economic, infrastructural, and social changes [1–4]. Many rapidly transitioning populations are Indigenous and/or marginalized, with transitions precipitated by structural and systemic processes such as colonialism, loss of land rights, and environmental degradation [3, 5–7]. Development schemes favoring urbanization and acculturation of subsistence-level and Indigenous groups have been justified and promoted because such changes often accompany increased access to modern healthcare and reductions in certain types of infectious disease, extreme poverty, and food insecurity, yet lifestyle change has simultaneously created an epidemic of cardiometabolic diseases in these populations around the world [3, 7–12]. Importantly, the details and drivers of these epidemics often differ: for example, political or economic pressure, legal land title, and desirability of Indigenous lands can influence the speed of their development and degradation [13]. Further, dietary shifts may depend on government policies (e.g. international trade involvement), market accessibility, and social practices [12, 14]. Additionally, while lifestyle transitions can be driven by external forces, individuals will also vary in how and how much they seek access to the market economy, formal education, healthcare, urban infrastructure, and other facets of lifestyle change.

Studies within individual countries or Indigenous populations exhibiting a gradient of subsistence-level to urban lifestyles suggest that subsistence living is protective against cardiometabolic diseases [15–21]. Poor diet, exposure to pollution, changes in sleep patterns, built infrastructure, market goods, and occupations that reduce daily activity levels in more urban environments are expected to drive these negative health effects [22–28]. Further, this body of work has generally shown greater effects of urban exposure on health outcomes in females than males [29]. Although focused studies of individual countries or Indigenous populations can be illuminating, we lack a generalized framework for quantifying lifestyle transitions and their effects on cardiometabolic health across populations. Building such a framework requires comparable, individual-level data across multiple transitioning populations and tools for summarizing these data for comparative research.

A major goal of evolutionary medicine has been to identify the specific lifestyle drivers underlying recent increases in cardiometabolic disease in transitioning communities. Defining and operationalizing lifestyle transitions is necessary to determine how different facets (e.g. market integration, acculturation, urbanization) relate and which are most predictive of health. For example, McDade and Adair [30] decomposed the urban environment into community-level versus individual-level factors, and later studies have investigated the influence of these, and other, lifestyle metrics on specific cardiometabolic health outcomes in individual populations (e.g. diet [31], physical activity [32], subsistence/wage labor reliance [33], material wealth vs. market access [21, 34–36], diet vs. material wealth [34], socioeconomic status vs. acculturation [37]). While impactful, this body of work is limited in its ability to establish a general framework for assessing which aspects of lifestyle variation most strongly affect cardiometabolic health in a comparative context.

Here, we investigate how measurements of urbanicity, industrialization, acculturation, and market integration affect cardiometabolic health by synthesizing data from two remote-living, subsistence-level Indigenous groups living in dramatically different ecological, economic, and sociopolitical contexts: the Turkana of northwest Kenya and Orang Asli of Peninsular Malaysia. Within the fields of global and public health, East Africa and Southeast Asia remain understudied relative to the Global North, and within their respective regions, the Turkana and Orang Asli are further underrepresented in public health, biomedical, and epidemiological studies [38]. We chose to work with these populations because they are experiencing rapid lifestyle transitions, and consequently, we were able to work with individuals of the same or similar genetic background spanning extreme gradients from traditional, subsistence lifestyles to fully industrialized and market integrated lifestyles [19, 39]. We use integrated and coordinated data collection to investigate three questions. First, we compare how facets of the lifestyle transition are occurring in Turkana and Orang Asli using ten scales–four adapted from previously developed scales and six new–to capture *a priori* domains of lifestyle variation (e.g. diet, built environment, material wealth) [19, 40, 41]. Second, we investigate the extent to which these domains are associated with 16 cardiometabolic health phenotypes. Specifically, we compare patterns between the two populations, use sensitivity analyses to identify the most important individual components of the top scales, and use piecewise analyses to further characterize the effects of lifestyle transition on cardiometabolic biomarkers. Third, we investigate whether these findings differ between females and males in both populations.

## METHODS

### Turkana

The Turkana are historically a nomadic pastoralist population living in the remote Turkana Basin in northwest Kenya. Traditionally, approximately 80% of the Turkana diet has been derived from animal products including milk, meat, and blood [19, 42], though these numbers vary highly depending on the season and geographic area. Ongoing infrastructure construction and rapid economic development of Kenya has resulted in the growth of several urban centers in and near traditional Turkana lands, the expansion of small-scale markets, and an increased reliance on agriculture. As a result, most Turkana no longer exclusively practice traditional pastoralism, instead relying on trade, small scale farming, and increased participation in the market economy [19, 43]. In addition to socioeconomic changes happening within the Turkana region, many Turkana have moved to highly urbanized areas in central Kenya in the last several decades, and now participate fully in the market-economy [19, 43]. Compared to subsistence-level and remote-living individuals, Turkana living in urban areas in central Kenya have higher body fat, cholesterol, and blood pressure; however, it remains unknown how cardiometabolic phenotypes are associated with different specific facets of the lifestyle gradient [19, 44].

### Orang Asli

The term ‘Orang Asli’ is an ethnonym referring broadly to the Indigenous peoples of Peninsular Malaysia (represented by at least 19 culturally distinct ethnolinguistic groups) with varied subsistence histories consisting of foraging, swidden agriculture, trade of rainforest products, or a combination thereof [45]. Orang Asli are multiethnic and typically categorized into three broad groups (the Negrito [Semang], Senoi, and Aboriginal Malay) who are genetically distinct but generally more genetically similar to each other than to ethnic Malays or other Asian populations [46]. Prior to the 1950’s, most Orang Asli lived in relatively small groups and relied primarily on subsistence economies. With rapid economic development in Malaysia, especially the expansion of rubber and oil palm agriculture, coupled with government programs aimed at integrating Orang Asli into the national economy, Orang Asli have been exposed to differing levels of lifestyle change [10, 47]. Today, this manifests as a dramatic lifestyle gradient within a relatively small country: some Orang Asli villages are surrounded by urban or peri-urban development while others consist of small camps of highly mobile individuals occupying interior rainforest regions with a continued heavy reliance on subsistence foraging and farming [39].

Sub-groups of the Orang Asli have been reported to have different levels of obesity and hypertension, but it remains unclear the extent to which identifiable aspects of lifestyle transitions directly influence these rates [48, 49].

### Cardiometabolic health biomarkers

**Figure 1:**
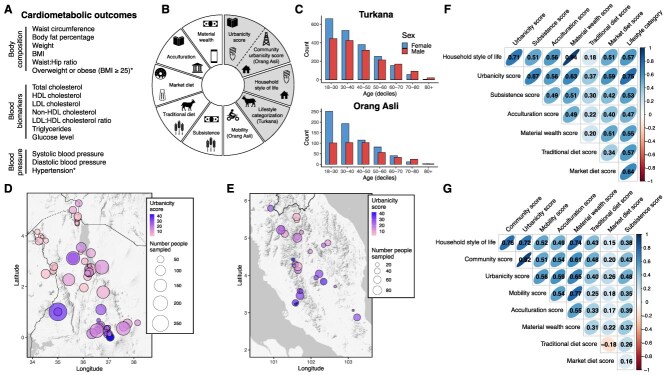
Study design overview. (A) The sixteen cardiometabolic traits we measured in both Turkana and Orang Asli. (B) Schematic of the ten scales used to quantify facets of lifestyle change. Scales adapted from prior studies are colored gray. The three scales used for only one population are denoted. (C) Age and sex distributions of all individuals in the study. Maps showing the location-based urbanicity score for each sampling location in (D) Kenya and (E) Malaysia, with points scaled to the number of individuals sampled in each location. Correlations between all lifestyle variation scales in the (F) Turkana and (G) Orang Asli.

The data used here are from two ongoing projects, the Turkana Health and Genomics Project (THGP) and the Orang Asli Health and Lifeways Project (OA HeLP), previously described in Lea *et al*. [19] and Wallace *et al*. [39], respectively. Data from both projects were cross-sectional. THGP data were collected between March 2018 and November 2023; data from OA HeLP were between June 2022 and December 2024 (*n* Turkana = 3691, *n* Orang Asli = 1119). As part of both projects, consenting adults (≥18 years) participated in structured surveys and the measurement of 14 cardiometabolic phenotypes, including waist circumference, body fat percentage, weight, BMI, waist-to-hip ratio, total cholesterol, high-density and low-density lipoprotein (HDL, LDL) cholesterol, non-HDL cholesterol, LDL-to-HDL ratio, triglycerides (mg/dL), glucose level (mg/dL), and systolic and diastolic blood pressure (mm Hg). We characterized hypertension (systolic blood pressure > 135 and diastolic blood pressure > 85), using the median values for ‘high-normal’ blood pressure from the most recent American Heart Association guidelines [50]. Overweight or obese (BMI ≥ 25) was determined using standard thresholds from the World Health Orangization [51] (Fig. 1A). Data were collected using highly similar methods for both studies and using standard protocols to facilitate comparison. Additional information is provided in the Supplemental Material.

### Development of scales to quantify particular facets of lifestyle variation

We quantified lifestyle variation in both populations using a total of ten scales which captured different facets of urbanicity, market integration, dietary transitions, and acculturation (hereafter ‘lifestyle scales’ or ‘scales’). Seven scales were replicated between the two populations, one was unique to Turkana, and two were unique to Orang Asli (Fig. 1B; Table S1). First, for both Turkana and Orang Asli, we adapted the previously published household style of life scale [H-SOL] from Gildner *et al*. [40], which measures an individual’s household size, building materials, and infrastructure (e.g. running water, electricity source). We also adapted the location-based lifestyle scale from Novak *et al*. [41], which captures population density, infrastructure available in the community, aggregate access to infrastructure (e.g. electricity source, sewage), material wealth, subsistence strategies, and education per location. We adapted this scale in two ways. We first generated a scale that maximized comparability between Turkana and Orang Asli (hereafter ‘urbanicity scale’ or ‘urbanicity score’; more details in Tables 1 and S1). Second, for Orang Asli, we had data on community-level attributes, and we generated a scale that maximized similarity to the original Novak *et al.* scale and term this the ‘community scale’ (more details in Tables 1 and S1). While very infrequent in our data, when locations were revisited, we generated distinct urbanicity scores per location, per year. For both H-SOL and the two scales adapted from Novak *et al*., we included items comparable to those from the original scale and, as possible, weighed items to recapitulate the original scoring structure. For Turkana, we also adopted a numeric version of the lifestyle categorization from Lea *et al*. [19], which characterizes Turkana-specific subsistence strategies.

**Table 1:** The ten lifestyle variation scales, the facets of lifestyle that each scale measured, and specific items included in each scale. Details of scale construction is in Table S1.

Scale	Domain of lifestyle targeted	Items included
Urbanicity scale (adapted from Novak *et al.* [41])	Population density and location-aggregate infrastructure and material wealth (as defined in ‘Western’ contexts)	Population density Proportion of houses with access to electricity, sewage Proportion of population engaged in subsistence practices Proportion of households with mobile phones, televisions Proportion of individuals >40 years with some education, proportion of individuals <40 years with some education
Community scale (adapted from Novak *et al.* [41]) (Orang Asli)	Population density, infrastructure available in the community at large, location-aggregate infrastructure and material wealth	Population density Proportion of houses with access to electricity, sewage Proportion of population engaged in subsistence practices Proportion of households with mobile phones, televisions Proportion of individuals >40 years with some education, proportion of individuals <40 years with some education Infrastructure available to access village (e.g. roads) Water, electricity, internet, and cell service infrastructure available in the community at large Time to nearest school, clinic, and hospital
Household style of life (H-SOL; adapted from Gildner *et al.* [40])	Household building materials and infrastructure	Household building materials Electricity, sewage, or tap water infrastructure Number of rooms in the house (Turkana only)
Material wealth	Extent of wealth as defined in ‘Western’ or industrialized contexts	Number of rooms in the house and number of household members per room (Turkana only) Household building materials Electricity, sewage, or tap/treated water infrastructure House has gas stove Ownership or access to mobile phone, TV, other material items
Acculturation	Extent of integration into majority culture of the country	Highest education level Languages spoken Religion (Orang Asli only)
Market-derived diet	Extent of market-derived foods incorporated in diet	Sugar, salt, and cooking oil consumption
Traditional diet	Extent (or lack thereof) of traditional foods consumed *this scale is coded such that higher values indicate less consumption of traditional foods	Turkana: meat, milk, blood consumption Orang Asli: rice, manioc, wild meat, and wild fish consumption
Subsistence	Extent of participation in subsistence-activities vs. wage labor	Occupation or subsistence activities engaged in
Mobility (Orang Asli)	Access to the market economy	Access to motorcycle or car Number of lifetime visits to Kuala Lumpur Number of visits to closest large town in last 2 weeks
Lifestyle categorization (adapted from Lea *et al.* [19]) (Turkana)	Delineation between Turkana living traditional, semi-traditional lifestyles, or highly market integrated/urban lifestyles	Turkana pastoralists defined as those solely practicing traditional pastoralism, relying on animal products, and eating traditional foods Peri-urban Turkana defined as those living in rural areas but not practicing traditional pastoralism Urban Turkana defined as those living in urban centers

We then developed five *a priori* scales to capture different key facets of lifestyle transition, including (i) material wealth, (ii) subsistence, which measures the extent to which individuals participate in wage labor vs. traditional subsistence activities, (iii) acculturation to the majority culture (e.g. speaking the majority language or practicing the religion of the predominant culture), and the extent to which individuals consume a (iv) traditional or (v) market-derived diet. Additionally, for Orang Asli only, we quantified individuals’ mobility and access to urban areas, as we expect that vehicle access in Malaysia is more indicative of market access than in Kenya given the size of the country (i.e. in Kenya, major urban centers are a multi-day drive from traditional Turkana lands). All scales are coded such that higher values indicate higher extent of urbanicity, acculturation, or market integration. All scales quantify lifestyle at the level of the individual except for the location-based urbanicity and community scales (which would be the same for all members of the same community).

While many of the lifestyle scales we generated have the same range (0–1), we do not expect that the same value across different scales necessarily represents the same point in the lifestyle transition (Fig. S1A–B). Further, we did not design these scales to represent the lifestyle transition in absolute terms. Instead, these scales were designed to capture relative variation in lifestyle features and be broadly applicable to multiple transitioning populations (Fig. S1A–B). After generating the lifestyle scales for each population, we compared the correlations between all scales for each population. Breakdowns of age, sex, and lifestyle are available in Tables S2 and S3. Items included in each scale are listed in Table 1, with further details in Table S1. Code to generate each scale is available on our GitHub (https://github.com/mwatowich/Multi-population_lifestyle_scales).

We also tested whether the items included in our ten scales could be decomposed into distinct axes of lifestyle variation. To do so, we used factor analysis, an unsupervised machine learning method, which is detailed in the supplement and was inspired by McDade and Adair [30]. Briefly, we performed factor analyses for the Turkana and Orang Asli separately, using all constituent items in the individual-level lifestyle scales we quantified for each population. We did not include items from the urbanicity or community scores as these were aggregated per location, nor did we include the Turkana-specific lifestyle categorization as it was not composed of individual numeric items.

### Analysis of lifestyle and cardiometabolic health

We used linear and generalized linear models to analyse continuous and binomial cardiometabolic outcomes, respectively, performing modeling separately for each population. For each scale, we computed variance explained by comparing the difference in R^2^ between models with and without the focal scale included. We then compared AIC between models that included each scale to predict a given cardiometabolic outcome. Setting the market diet scale as our focal scale, because we hypothesized that this scale would best predict variance in most cardiometabolic biomarkers, we compared the AIC values from all models to the AIC value from the market diet scale and found the difference between AIC values; we did this for each cardiometabolic outcome. We then found the mean of the difference in AIC values for each lifestyle scale, across all cardiometabolic biomarkers, biomarkers related to body composition, biomarkers related to blood pressure, and biomarkers related to blood lipids. To understand whether lifestyle scales performed similarly among females and males, we repeated the modeling approach described above for these sexes individually. We then explicitly tested whether cardiometabolic phenotypes in females and males were differentially impacted by lifestyle variation by modeling sex by lifestyle interactions for each scale.

For the three scales that best predicted cardiometabolic health in each population in the sex-combined analyses, we performed sensitivity analyses to determine the importance of specific contributing terms. In these sensitivity analyses, we removed one item in the scale and compared the difference in R^2^ between the model with the complete scale and the scale calculated with the item removed.

To quantify whether the top factors from the factor analysis were more strongly predictive of cardiometabolic health than the lifestyle scales we generated a priori, we repeated the modeling approach described above between the top three scales that best predicted cardiometabolic health in each population and the top two factors from our factor analysis.

We performed a Benjamini-Hochberg correction for multiple hypothesis testing for each analysis using the *p.adjust* function in R. We performed all analyses using the R computing language and RStudio version 4.3.2 [52].

## RESULTS

### Changes in diet and built environment are decoupled during lifestyle transitions

We first generated ten scales (seven in both populations, one only in Turkana, two only in Orang Asli) that encapsulated different facets of the lifestyle transition during urbanization and industrialization (Tables 1 and S1; Figs. 1B and S1). These analyses revealed that individuals span a wide lifestyle gradient in both countries (Figs. 1C–E and S1), and that different facets of lifestyle covary in distinct ways (Fig. 1F–G). In particular, as the availability of highly processed foods generally occurs before larger-scale changes in infrastructure, occupation, education, and the accumulation of material wealth, we hypothesized that scales reflecting non-dietary facets of urbanicity would be more correlated to each other than to scales quantifying diet [25,31]. Indeed, scales capturing non-dietary lifestyle features such as the built environment, material wealth, and acculturation were more correlated to one another (mean Pearson r = 0.59 [Turkana], mean r = 0.57 [Orang Asli]) than to scales quantifying traditional or market-derived diet (mean r = 0.40 [Turkana], mean r = 0.24 [Orang Asli]) (*t*-test *P* = 1.60 x 10^−3^ [Turkana], *t*-test *P* = 6.84 x 10^−7^ [Orang Asli]; Fig. 1F–G). Overall, lifestyle measures in Orang Asli trended towards being less correlated than in Turkana (*t*-test *P* = .12); this suggests that there may be more heterogeneity in how lifestyle is changing in Peninsular Malaysia than Kenya, or that greater cultural heterogeneity among Orang Asli contributes to more variation in adoption of lifestyle changes.

### Urbanicity is strongly associated with cardiometabolic health

**Figure 2:**
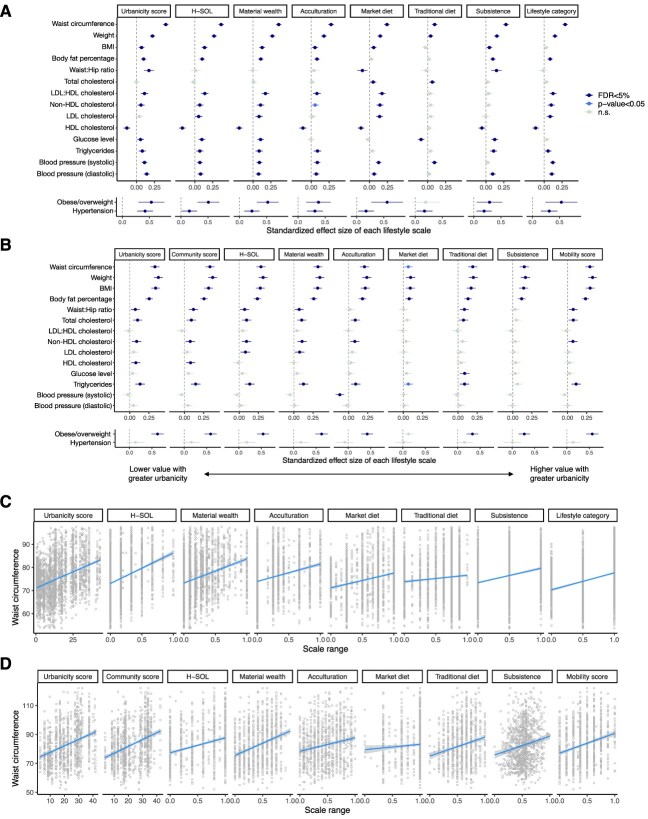
Effect of lifestyle on sixteen cardiometabolic health outcomes for both populations. Standardized effect sizes of lifestyle–as quantified by each of the ten scales–for all cardiometabolic traits in (A) Turkana and (B) Orang Asli. Dark blue points represent associations which passed an FDR of 5%, medium blue points represent associations with a significant *P*-value <.05 but that did not pass a FDR of 5%, and light blue points represent non-significant associations. Waist circumference is positively associated with higher urbanicity as quantified by nearly all scales in (C) Turkana and (D) Orang Asli.

Next, we sought to understand which features of lifestyle most strongly predicted (i.e. yielded best model fit) our 16 cardiometabolic health phenotypes. As expected, we found that less traditional lifestyles (e.g. higher urbanicity, acculturation, integration to the market economy) were associated with larger waist circumference, higher weight, higher BMI, higher body fat percentage, higher triglycerides, and greater prevalence of obesity or overweight in both the Turkana and Orang Asli (Fig. 2A–D; Tables S4–S5). Among the Turkana, we also observed that more urban lifestyles were positively associated with higher blood pressure, higher LDL:HDL cholesterol, and higher glucose levels (Fig. 2A; Table S4). We also re-ran these analyses within each of the three major Orang Asli sub-groups and found similar results to those including all Orang Asli in a single analysis (Pearson’s correlation of within-group effects to overall Orang Asli dataset: r_Senoi_ = 0.61, *P* = 1.84 x 10^−14^, r_Aboriginal Malay_ = 0.64, *P* = 6.98 x 10^−16^, r_Negrito_ = 0.86, *P* = 2.2 x 10^−16^) (Fig. S2A–B). As expected, we observed substantial heterogeneity in the amount of variance explained by lifestyle per cardiometabolic biomarker, with traits related to body composition being more highly explained by lifestyle than those related to blood pressure or lipids (Fig. S3A–B).

**Figure 3:**
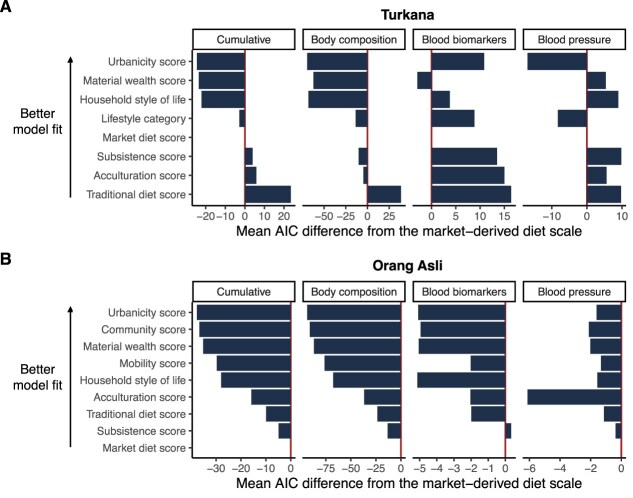
Scales that measure infrastructure and ‘Western’ material wealth better predict cardiometabolic biomarkers than scales that measure acculturation, subsistence/occupation, or diet. The mean difference in AIC from the market-derived diet scale across all cardiometabolic traits (cumulative), and traits associated with body composition, blood biomarkers, or blood pressure in (A) Turkana and (B) Orang Asli. The y-axis is ordered by lowest mean AIC across the four categories shown.

To determine which lifestyle scale explained the greatest amount of variance, we compared model fits for each cardiometabolic trait. We found that two lifestyle scales–the urbanicity score and material wealth scale–consistently best predicted cardiometabolic phenotypes and found strikingly high concordance between results for both populations (Fig. 3A–B; Tables S4–S5). Both of these scales primarily focus on material wealth and infrastructure. When we assessed categories of cardiometabolic traits, we found that body composition phenotypes in Turkana and Orang Asli were best predicted by our urbanicity and material wealth scales. Turkana blood biomarkers (i.e. lipids) were best predicted by our material wealth and market diet scales, while blood biomarkers in Orang Asli were best predicted by the urbanicity, material wealth, and H-SOL scales. Blood pressure in Turkana was best predicted by urbanicity score and lifestyle category, while blood pressure in Orang Asli was best predicted by our acculturation scale (Fig. 3A–B). Interestingly, we observed similarities in the scores that were generally least predictive in both populations, the diet-related and the subsistence activity scores (Fig. 3A–B; Tables S4–S5).

### Factor analyses show that the built environment and diet act independently on cardiometabolic health across populations

**Figure 4:**
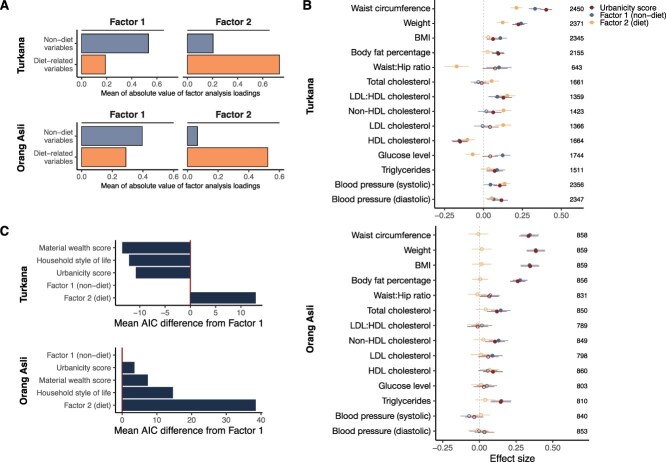
Factor analysis decomposes lifestyle into two axes: infrastructure and the built environment vs. diet. (A) Loadings for factors 1 and 2 in both populations capture highly similar features of the data, with factor 1 generally correlating with features of lifestyle not related to diet and factor 2 being most associated with diet-related variables. (B) Standardized effect sizes showing the effect of each factor (factors modeled separately) on each cardiometabolic trait. Effect sizes from models of the location-based urbanicity scale are shown for a comparison. Filled circles indicate statistically significant effects (FDR < 5%) and open circles are not statistically significant. Sample sizes are shown on the right-hand side. (C) Mean difference in AIC across all continuous cardiometabolic traits for the top three lifestyle scales and the two factors from our factor analysis.

We next asked whether the items included in our lifestyle scales decomposed onto distinct facets of the lifestyle transition using an unsupervised machine learning method. We determined that two factors accounted for the majority of the variance (factor 1 proportion of variance: 36% [Turkana], 35% [Orang Asli], factor 2 proportion of variance: 25% [Turkana], 19% [Orang Asli]) and were strikingly consistent between the two populations: the first factor was primarily associated with features related to infrastructure (e.g. electricity, plumbing) and the built environment (e.g. housing materials, material wealth) (Figs. 4A and S4A–F). The second factor loaded most strongly onto features related to diet and was highly associated with the *a priori* market diet scale in both Turkana (r = 0.97) and Orang Asli (r = 0.95) (Figs. 4A, S4B,D–F). We then modeled associations between each factor and the 16 cardiometabolic phenotypes and generally found expected relationships, for example that body composition measures were positively and additively associated with both factors for each population (Fig. 4B; Tables S6–S7). Together, these results recapitulated our findings from *a priori* defined lifestyle scales: features associated with the urban built environment are more predictive of cardiometabolic health than diet.

We also asked whether the factors described above were more strongly predictive of cardiometabolic health than our *a priori* measures of lifestyle. To this end, we compared models including factor 1, factor 2, or each of the top three performing lifestyle scales in each population (material wealth, H-SOL, and the urbanicity scale). In Turkana, models with the three lifestyle scales had lower AIC scores across all health outcomes than either factor 1 or factor 2, with material wealth and H-SOL being the best performing measures and performing very similarly (Fig. 4C; Table S6). For Orang Asli, factor 1 and the urbanicity scale were the top performing measures and performed very similarly; these were followed by H-SOL, the material wealth score, and finally, factor 2 (Fig. 4C; Table S7).

### Female body composition is often more strongly impacted by urbanicity than male body composition

**Figure 5:**
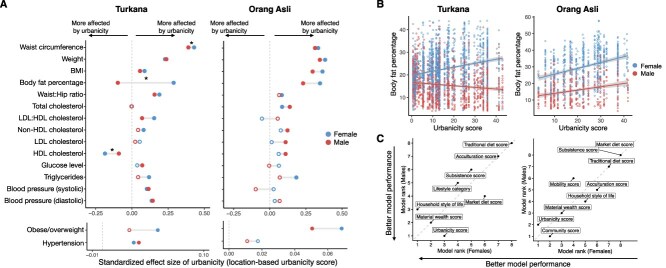
Female body composition is generally more impacted by urbanicity than males. (A) Standardized effect size of urbanicity from models within females (blue) and males (red) for both populations. Points further from y = 0 indicates a stronger effect of urbanicity. Stars denote significant sex x lifestyle interaction effects from the interaction models. Filled circles denote effects statistically significant at a FDR < 5%, open circles are not significant at FDR < 5%. (B) Example of interactive effects of sex and urbanicity (significant in Turkana, trending in Orang Asli). (C) Comparison of rank order of mean model fit between males and females across all cardiometabolic traits in both populations.

We were interested in understanding whether (i) lifestyle variation affects cardiometabolic health in a sex-dependent manner and (ii) whether the measures of lifestyle that best predict cardiometabolic phenotypes are consistent between these sexes. We observed that many cardiometabolic phenotypes exhibited significant sex-dependent effects, especially among Turkana (Figs. 5A and S5A–B; Table S8). For example, focusing on the urbanicity scale, we found three significant sex by lifestyle effects in Turkana and that cardiometabolic phenotypes were more strongly affected by urbanicity in females than males for 10 of 16 traits, especially for traits related to body composition (5 of 6; Figs. 5A–B and S5A; Table S8). In Orang Asli, many cardiometabolic traits showed similar trends of sex by lifestyle effects but were not significant following multiple hypothesis testing correction (Figs. 5A and S5B; Table S9). Additionally, we found that the rank-order of lifestyle scales was highly similar between the sexes in both populations, with the material wealth and urbanicity scales best predicting cardiometabolic health in both sexes (Fig. 5C; Tables S10–S11). In both populations, scales measuring subsistence-participation and traditional diet were among the least predictive scales for cardiometabolic health in both sexes (Fig. 5C; Tables S10–S11).

## DISCUSSION

Indigenous peoples around the world have an average life expectancy approximately 10 years shorter than non-Indigenous individuals, largely due to non-communicable and cardiometabolic diseases, many of which increase with the transition from subsistence lifestyles to urban or market integrated lifestyles [3, 53]. Reducing health gaps for subsistence-level and Indigenous populations and understanding the lifestyle drivers most substantially contributing to declining cardiometabolic health in urban, industrialized populations is a major goal of evolutionary medicine and public health researchers, healthcare practitioners, and policy-makers worldwide [3]. It is possible to conceive of many ways that changing lifestyles influence cardiometabolic health: possible mechanisms range from economic forces affecting physical activity, to altered dietary options, ethnic non-retention and acculturation, or features of the urban built environment and modern amenities that broadly shift behavioral patterns [15, 17–19, 54]. Yet, few studies have attempted to directly compare different dimensions of lifestyle change in a way that could be feasibly applied in cross-cultural settings–a pressing necessity given the rapid pace of lifestyle change in subsistence-level groups worldwide. By integrating detailed anthropological and biomarker data from two transitioning subsistence-level, Indigenous societies, we demonstrate that lifestyle variation can be (i) meaningfully quantified in multiple dimensions that separate people living across wide gradients, (ii) measured empirically such that cross-cultural comparison is feasible, and (iii) linked directly with physiological data to determine how dimensions of lifestyle differentially impact health.

First, we found that despite lifestyle transitions manifesting very differently in Kenya and Peninsular Malaysia, lifestyle factors exhibit strikingly similar effects on cardiometabolic health. Together, these results show that quantitative measures of lifestyle are practically useful in distinct contexts and may be generally useful for understanding the drivers of cardiometabolic decline, identifying populations most vulnerable to urbanization, or designing intervention approaches, even across disparate environmental and socio-political contexts. Further, the structural and systemic reasons underpinning Indigenous peoples’ lifestyle change are diverse, from autonomous decisions to pursue wage labor or urban economic opportunities to forced resettlement and assimilation [3, 55]. The lifestyle change experienced by Turkana and Orang Asli encompasses both of these reasons [10, 19, 43], but nonetheless we again observe similar overall effects of lifestyle variation on health.

Second, we found that the built environment and material wealth more strongly predicted cardiometabolic health on average than measures of seemingly more proximate features, such as diet. Interestingly, our factor analyses suggested that these aspects of lifestyle change are commonly separable and can thus be separately targeted for example for monitoring or intervention. Accumulating evidence from multiple subsistence-level populations around the world suggests similar findings, for example by Kraft *et al*. [17] in the Tsimane of Bolivia, Liebert *et al*. [34] in the Shuar of Ecuador, and Mattison *et al*. [4] in the Mosuo of China. As processed foods and market-derived additives are well established to have direct effects on blood lipid levels and adiposity [56, 57], we expect that our results of strong effects of urban infrastructure reflect that individuals living in these areas (e.g. with roads, gridded electricity) have likely had access to market integrated items for longer and/or in at higher quantities than individuals with less urban infrastructure. In support, we find that Orang Asli exposed to higher urbanicity–who generally consume more processed foods–report significantly less change in the consumption of processed oil and sugar between adulthood and childhood (oil: β = −0.46, *P* = 8.74 x 10^−6^, sugar: β = −0.48, *P* = 4.32 x 10^−6^).

Third, we find that female cardiometabolic health–especially body composition–was more affected by urbanicity than that of males, recapitulating previous findings in Turkana and other populations, and highlighting an important intervention target [3, 19, 29]. One potential explanation is greater sex difference in activity levels with increasing urbanicity, with females reducing their average activity levels to a greater extent than males in many transitioning societies, especially in areas that become less walkable [22, 58]. Reduced activity levels and increased time sedentary likely contribute to urban adiposity and cardiometabolic morbidity more generally in LMIC [23, 58]. To fully address these gaps, in-depth anthropological research is needed to understand sex-differences in activity levels in traditional, subsistence-level societies and how sex-based social roles and activity levels change throughout the lifestyle transition.

Practically, our study offers several methods to quantify diverse facets of lifestyle at the individual- and community-levels. Public and Indigenous health professionals, anthropologists, and biomedical researchers are increasingly working across populations and countries, and methods that perform robustly in different contexts will help inform health monitoring and intervention strategies. Further, the scales we test that captured infrastructure and material wealth using 10 short survey questions performed better or comparably to the products of a factor analysis that included dozens of items (Table S12). We therefore suggest that cross-cultural research would benefit in terms of time and resource efficiency from adopting the urbanicity, material wealth, and H-SOL scales developed here. As studies with Indigenous and subsistence-level communities are often in remote locations with limited resources, it may in some cases be beneficial to collect aggregate location-level data based on a representative sample of individuals rather than data for each individual. Capturing general environmental features of communities may also be less prone to respondent error or recall about individual-level behavior.

## LIMITATIONS

Our study has limitations. First, we acknowledge that we use several outcome measures that may have different relationships to morbidity or mortality in our participant populations relative to the high-income contexts in which they have been validated and in which they are commonly used, namely continuous BMI, obesity (yes/no), and hypertension (yes/no) [59]. Second, we acknowledge that there is mixed evidence that BMI under 30 (i.e. the threshold for obesity categorization) negatively impacts health [60, 61]. As BMI is systematically biased by height [62], comparisons of BMI between populations with substantially different population-average heights is not ideal. However, within-population BMI comparisons can be useful as a coarse measure of body composition, and we include BMI in our analyses because it has been used in many previous studies and allows our results to be compared to prior work. Third, we note that while many of the lifestyle scales we present here have the same range, these scales are relative representations of lifestyle transition and values will change depending on the items included in each scale. Scales may need to be adjusted to reflect traditional practices which differ between populations, for example quantifying pastoralism vs. farming and foraging in the two populations we present here. The scales we present do not necessarily quantify equivalent positions in the lifestyle transition but instead are useful in contexts in which there is variation across the lifestyle gradient and/or between populations. Fourth, both diet scales were based on participants’ recall of average weekly consumption of particular foods, which may be more prone to respondent error than more static environmental features. Given the challenging nature of measuring diet from participant recall and that diet likely affects cardiometabolic health through both the type and frequency and amount of intake it is possible that greater error in these measures partially accounts for their lower predictive power of cardiometabolic health. Finally, while studying two unrelated populations that live in very different environments provides strong evidence for the generalizability of our findings, repeating this investigation in additional transitioning populations will further illuminate the factors that drive context-dependency of lifestyle effects on cardiometabolic health.

## CONCLUSIONS

In conclusion, we find a diverse set of 16 cardiometabolic phenotypes were similarly affected by lifestyle features in two very different Indigenous groups spanning extreme lifestyle gradients, and that infrastructure and material wealth exerted stronger effects on cardiometabolic health than diet in both populations. While we did not formally investigate additional proximate factors that might moderate the relationship between lifestyle change and cardiometabolic health, the extensive variation we observe in health among individuals with similar lifestyles suggests that many factors join to determine inter-individual variation. Future work could identify the individual-level factors that predict vulnerability versus resilience during lifestyle transitions, for example physical activity levels [22, 54, 58], sleep quality [63], early life experiences [64], and social connectedness and other sources of social protection [65]. Additionally, while our study was cross-sectional, one compelling future use of validated, generalizable scales such as these is the ability to quantify longitudinal changes in urbanization, industrialization, market-integration, and acculturation occurring within communities; understanding within-individual rates of change in lifestyle and health would provide a powerful framework for identifying causal factors and exploring non-linear relationships. In summary, our work provides public health researchers a unified framework for quantifying different facets of the lifestyle transition that is comparable across populations, and our findings highlight that lifestyle change–especially increased urbanization–has extensive effects on cardiometabolic health across populations, contexts, sexes, and is a major target for intervention.

## Supplementary Material

Supplementary_material_eoag007

## DATA SHARING

Our highest priority, and that of the Turkana Health and Genomics Project and the Orang Asli Health and Lifeways Project, is to minimize risk to study participants. As such, both projects adhere to the ‘CARE Principles for Indigenous Data Governance’ (Collective Benefit, Authority to Control, Responsibility, and Ethics) and are committed to the ‘FAIR Guiding Principles for scientific data management and stewardship’ (Findable, Accessible, Interoperable, Reusable). To adhere to these principles while minimizing risks, individual-level data are stored in the protected data repositories and are available through restricted access. Requests for de-identified, individual-level data should take the form of an application that details the exact uses of the data and the research questions to be addressed, procedures that will be employed for data security and individual privacy, potential benefits to the study communities, and procedures for assessing and minimizing stigmatizing interpretations of the research results. Requests for de-identified, individual-level data will require institutional IRB approval (even if exempt). THGP and OA HeLP are committed to open science and the project leadership is available to assist interested investigators in preparing data access requests (see turkanahgp.com and orangaslihealth.org for further details and contact information). Data needed to evaluate the conclusions in the paper are present in the paper, the Supplementary Materials, and deposited on Zenodo (DOI 10.5281/zenodo.15684153). Code used to generate lifestyle scales and to perform analyses described herein is available on our GitHub (https://github.com/mwatowich/Multi-population_lifestyle_scales).
